# Primary segmental omental torsion, mimicking acute appendicitis

**DOI:** 10.25122/jml-2023-0429

**Published:** 2024-01

**Authors:** Paschalis Gavriilidis, Salomone Di Saverio, Mauro Podda, Nicola de’Angelis

**Affiliations:** 1 Department of Surgery, Saint Helena General Hospital, Jamestown, UK; 2 Department of Surgery, San Benedetto del Tronto Hospital, San Benedetto del Tronto, Italy; 3 Department of Surgical Science, University of Cagliari, Cagliari, Italy; 4 Colorectal and Digestive Surgery Unit, Beaujon Hospital, Clichy, France

**Keywords:** primary omental torsion, hemoperitoneum, torsion

## Abstract

Primary segmental omental torsion (PSOT) is a very rare cause of acute abdominal pain, and it may often imitate the clinical picture of acute appendicitis. In instances of acute abdominal pain without anorexia, nausea, and vomiting, omental torsion should be included in the differential diagnosis. Any misdiagnosis may lead to major complications such as intraabdominal abscesses and adhesions. A 63-year-old overweight man with a body mass index (BMI) of 41 Kg/m^2^ presented to the emergency department on a remote island with acute abdominal pain. His medical history included type 2 diabetes mellitus managed with insulin, essential hypertension, osteoarthritis, and no previous abdominal operations. He reported a sharp pain originating in the epigastrium and the right hypochondrium that started five days prior. Physical examination revealed rebound tenderness and guarding across the abdomen with a positive McBurney sign. However, the patient did not report vomiting and was not nauseous. Vital signs were as follows: blood pressure 116/56 mmHg, heart rate 98 beats/min, respiratory rate 19 breaths/min, and a temperature of 38.2 ^0^C. Laboratory results showed a white blood cell count of 10.6, neutrophils of 8.11, C-reactive protein (CRP) 74 mg/l, haemoglobin11.6 g/dl, and hematocrit 36.9%. Due to the absence of a radiographer at the hospital during that period, no imaging investigations were conducted. Diagnostic laparoscopy demonstrated diffused hemoperitoneum and necrotic mass at the site of the hepatic flexure. Initially suspected to be an advanced colon cancer, the decision was made to proceed with open surgery. The necrotic segment of the omentum was found at the right superior point of attachment of the omentum to the hepatic flexure. Consequently, the necrotic segment of the omentum was resected. A thorough investigation of the abdominal cavity did not detect any other abnormalities or pathologies. The patient recovered uneventfully and was transferred to the surgical ward. Torsion of the omentum is a very rare cause of acute abdominal pain. This case highlights the necessity of considering PSOT in the differential diagnosis of acute abdominal pain, especially in cases where symptoms are suggestive of appendicitis but diagnostic findings are negative.

## INTRODUCTION

The first documented case of primary omental torsion was reported by Eitel in 1899 [1]. Since this initial report, fewer than 300 cases have been described in the literature so far [2,3]. Primary omental torsion, defined by the twisting of an omental segment along its longitudinal axis without accompanying intra-abdominal pathology, is characterized by two main macroscopic features: a narrow neck and a necrotic segment [1-3]. This condition can present as either monopolar, with its southern pole free, or bipolar, in secondary cases, where both poles are attached to either a pathological condition or adhesions [4-7]. Primary omental torsion is more frequent in the right part of the omentum than the left due to its greater mobility, length, and weight [4-7]. Predisposing factors include bifid omentum, accessory omentum, and tongue-like projection from the free edge of the omentum. In addition, an uneven distribution of fat within the omentum in cases of morbid obesity predisposes individuals to omental torsion and, consequently, to venous stasis, thrombosis, and necrosis [5-7]. It has been reported that almost 80% of cases present with right lower abdominal pain and may imitate acute appendicitis, cholecystitis, and bowel perforation [8,9]. The preferred diagnostic method is a CT scan. Principal characteristics are the whirling pattern of the affected part of the omentum and hyper-attenuated streaks of fat beneath the parietal peritoneum in the affected segment [6,9,10]. However, most cases are diagnosed intraoperatively, and the indicated treatment is surgical excision [3,4,9].

The aim of this case report was to present the management of primary omental torsion on a remote island without the help of preoperative imaging.

## CASE REPORT

A 63-year-old man presented to the emergency department of a hospital on a remote island hospital with acute abdominal pain. Past medical history included essential hypertension, type 2 diabetes mellitus managed with insulin, osteoarthritis, and morbid obesity (BMI: 41 Kg/m^2^). Vital signs at admission were blood pressure 116/56 mmHg, heart rate (HR) 98 beats/min, respiratory rate 19 breaths/min, temperature 38.2 ^0^C. Laboratory results were: white blood cell count 10.6 (normal range 4.0–11.0), neutrophils 8.11 (normal range 2.5–6.0), C-reactive protein (CRP) 74 mg/l (normal range 0–9), hemoglobin 11.6 g/dl (normal range 13.0–18.0), hematocrit 36.9% (normal range 40–54). Physical examination revealed a positive McBurney point, rebound tenderness, and involuntary guarding across the lower abdomen. Due to the absence of a radiographer during that period, further investigation with a CT scan was not feasible.

**Figure 1:**
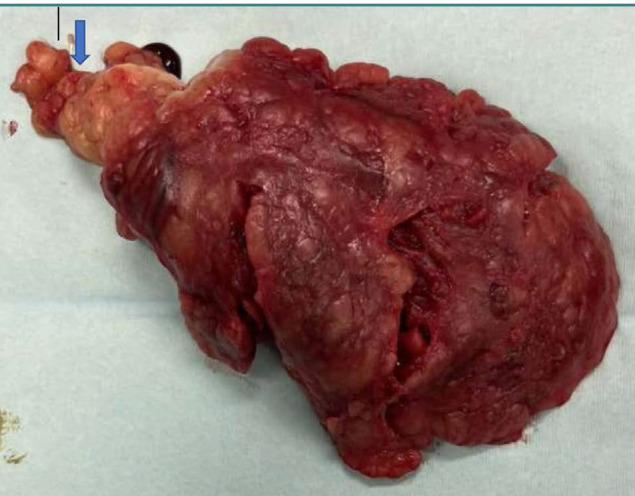
Surgical specimen of primary segmental omental torsion. The arrow indicates the twisted neck of the segment of the omentum.

The patient consented to undergo diagnostic laparoscopy, with the option to proceed based on findings. The presence of hemorrhagic fluid in the abdominal cavity raised suspicion for a distinct pathology from appendicitis. A solid hemorrhagic mass was identified in the right hypochondrium and on the site of the hepatic flexure. The rest of the abdominal organs were normal. Given the potential for the mass to represent a colonic tumor, the decision was made to transition to open surgery. A twisted segment of the omentum was found and subsequently resected (Figure 1). A thorough examination of the abdominal cavity revealed no anatomical abnormalities or additional pathologies. The patient recovered uneventfully.

## DISCUSSION

Primary omental torsion is a very rare disease, often mimicking the clinical presentation of acute appendicitis. The reported incidence rate is 0.0016% to 0.37%, and compared to appendicitis, it has a ratio of less than 4 cases per 1000 cases of acute appendicitis [11-13]. Considering that the current population of Saint Helena is 5,493 (2019 estimate), this makes our case more interesting [11-16].

This rare disease should be considered in the differential diagnosis when the clinical picture of the acute appendicitis is not typical. Usually, the primary symptom of omental torsion is the acute onset of sharp pain non-radiating elsewhere [14]. In our case, the patient reported sharp pain at the epigastrium five days prior. This symptom is not compatible with the onset of acute appendicitis. Usually, patients with primary omental torsion do not present with anorexia, nausea, and vomiting. In addition, inflammatory markers might be normal in most cases [15]. In our case, the patient did not complain of nausea or vomiting. However, although the patient's white blood cell count was normal, indicating no general inflammatory response, the CRP levels were significantly elevated, highlighting an acute inflammatory process.

Given that primary omental torsion may present with general symptoms associated with acute abdominal pain, it should be considered in the differential diagnosis of other diseases. These include appendicitis, cholecystitis, caecal diverticulitis, perforated duodenal ulcer, bowel obstruction, ectopic pregnancy, ovarian cyst torsion, salpingitis, Meckel diverticulum, mesenteric adenitis and accessory spleen [13,17,18]. A significant risk factor for primary omental torsion is the uneven distribution of fat in individuals with morbid obesity [4-7]. Therefore, in cases of morbid obesity, as it was our patient, the diagnosis of morbid obesity should be included in the differential diagnosis.

It has been reported that only 0.6% to 4.8% of cases of primary omental torsion are diagnosed preoperatively [19]. There is a dilemma in these cases whether to treat them conservatively [20]. Conservative treatment includes anti-inflammatory medications, prophylactic antibiotics, and analgesics. However, there is an increased risk of major early and late complications, such as intraabdominal abscesses and adhesions induced by the persistence of necrotic tissue in the abdominal cavity [19,20].

In the majority of cases published so far, open surgical excision was the treatment of choice [21]. However, after the invention of laparoscopy and considering all the benefits of minimal access surgery, laparoscopic options can be considered the treatment of choice [21,22]. Emergency laparoscopic procedures can be categorized into therapeutic and diagnostic types. In the therapeutic scenario, a specific pathology is assumed following a specific laboratory and imaging diagnostic workup. Consequently, a specific laparoscopic surgical procedure is planned and performed. In contrast, in the diagnostic scenario, the imaging workup failed to determine the cause of the abdominal emergency, or as it was our case, there was no adequate technical support to further investigate the patient. Here, the primary goal of laparoscopy is to identify the underlying cause of the acute abdominal condition. The effectiveness of diagnostic laparoscopy is significant, with a success rate in achieving a definitive diagnosis ranging between 86 and 100% [23,24]. This method is recommended for patients with persistent, severe symptoms or significantly abnormal laboratory results, underscoring its importance in the diagnostic process [25].

## CONCLUSION

Primary omental torsion is a rare disease that presents with generic symptoms and signs of acute abdominal pain. Usually, it is diagnosed intraoperatively during diagnostic laparoscopy. The potential for misdiagnosis is particularly concerning in remote locations, such as remote islands, and could have serious consequences.

## Data Availability

The authors declare that data supporting the findings of this study are available within the article. A preprint has been previously published in researchgate.net (Gavriilidis P, *et al*. Preprint August 2022).
